# Liver Sinusoidal Endothelial Cells Escape Senescence by Loss of p19ARF

**DOI:** 10.1371/journal.pone.0142134

**Published:** 2015-11-03

**Authors:** Petra Koudelkova, Gerhard Weber, Wolfgang Mikulits

**Affiliations:** Department of Medicine I, Division: Institute of Cancer Research, Comprehensive Cancer Center, Medical University of Vienna, Vienna, Austria; University of Medicine, Greifswald, Germany, GERMANY

## Abstract

Liver sinusoidal endothelial cells (LSECs) represent a highly differentiated cell type that lines hepatic sinusoids. LSECs form a discontinuous endothelium due to fenestrations under physiological conditions, which are reduced upon chronic liver injury. Cultivation of rodent LSECs associates with a rapid onset of stress-induced senescence a few days post isolation, which limits genetic and biochemical studies *ex vivo*. Here we show the establishment of LSECs isolated from p19^ARF-/-^ mice which undergo more than 50 cell doublings in the absence of senescence. Isolated p19^ARF-/-^ LSECs display a cobblestone-like morphology and show the ability of tube formation. Analysis of DNA content revealed a stable diploid phenotype after long-term passaging without a gain of aneuploidy. Notably, p19^ARF-/-^ LSECs express the endothelial markers CD31, vascular endothelial growth factor receptor (VEGFR)-2, VE-cadherin, von Willebrand factor, stabilin-2 and CD146 suggesting that these cells harbor and maintain an endothelial phenotype. In line, treatment with small molecule inhibitors against VEGFR-2 caused cell death, demonstrating the sustained ability of p19^ARF-/-^ LSECs to respond to anti-angiogenic therapeutics. From these data we conclude that loss of p19^ARF^ overcomes senescence of LSECs, allowing immortalization of cells without losing endothelial characteristics. Thus, p19^ARF-/-^ LSECs provide a novel cellular model to study endothelial cell biology.

## Introduction

Endothelial cells (ECs) represent a unique cell population originating from the mesoderm and lining all vessels in the organism. ECs can be divided into blood and lymphatic ones based on the capillary surface they are forming. From large vessels to small capillaries, ECs form compact monolayers with semi-permeable properties, mediating transport of small metabolites, migration of immune cells and vessel tone [1, 2]. Liver sinusoidal endothelial cells (LSECs) represent a subpopulation of non-parenchymal cells in adult liver [3]. In comparison with other ECs, they possess a unique morphology due to multiple membranous pores, called fenestrations, which are organized into sieve plates [4]. Due to their size of about 0.2 μm, fenestrations mediate fast transfer of small molecules, which accelerates metabolic exchange under controlled selection.

Platelet endothelial cell adhesion molecule (PECAM-1), also known as CD31, is considered as golden standard marker of endothelial cells [5, 6]. Unfortunately, many conflicting reports refer to CD31 expression in LSECs *in vivo* and *in vitro* [7–10]. Interestingly, March et al. showed that CD31 expression is predominantly high in the central and portal endothelium of rat liver and low expression is detected in liver sinusoids [10]. CD31 expression could be barely detected in freshly isolated LSECs which correlated with *in vivo* data. However, CD31 expression increased in cell culture at day 3 after defenestration, probably being accompanied by loss of endothelial phenotype. While CD31 expression is contradictorily published on ECs, the expression of vascular endothelial growth factor receptor 2 (VEGFR-2) is almost exclusively restricted to ECs [11]. In liver, VEGFR-2 expression can be exclusively detected on LSECs [12], thus belonging to markers which enable to distinguish ECs from other liver cell populations [13]. In addition, ECs are known to express several types of cadherins, including vascular endothelial (VE)-, P-and N-cadherin that are part of adherens junctions [14], von Willebrand factor (vWF) [15], stabilin-1 (Stab-1) and Stab-2 [16], CD32b [17], CD146 [9], and the lymphatic vessel endothelial hyaluronan receptor (Lyve)-1 [17].

Human umbilical vein endothelial cells (HUVECs) are frequently used as an EC model in vascular biology as these cells proliferate in cell culture with limited cell doublings [18]. However, conclusions drawn from experiments using HUVECs have to be carefully interpreted in organ-specific studies due to the wide heterogeneity among different types of endothelium. For multiple reasons, tissue-specific ECs are beneficial as these cells can be investigated in a homotypic setting, allowing more accurate results [19]. Yet, organ-specific ECs are hardly available. While human hepatic sinusoidal endothelial cells (HSECs) can be propagated in cell culture for 7–8 passages [15], their handling is delicate as they do not overcome several freeze-thaw cycles. Even more challenging, rodent LSECs [10] cannot be propagated after liver perfusion as they die within few days after cultivation.

The lifespan of human primary cells *in vitro* is affected by telomere length and their shortening during cell division. When telomeres reach critical length, cells enter mitotic crisis and as protection from further division, they undergo replicative senescence. In contrast, murine cells have active telomerase and are protected from the senescence induced by telomere shortening [20], yet their proliferative capacity remains finite due to enhanced expression of negative cell cycle regulators p16^INK4a^, p21^Cip1^, p53 or its regulator p19^ARF^ [21]. The INK4a/ARF locus encodes for the two crucial tumor suppressor proteins p16^INK4a^ and p19^ARF^ (p14^ARF^ in humans) which act upstream of the retinoblastoma and Mdm2/p53 pathways, respectively [22]. p19^ARF^ binds to Mdm2, a negative regulator of p53, thus stabilizing it and allowing p53 to act as a tumor suppressor responsible for cell cycle arrest and apoptosis [23, 24]. Mice lacking p19^ARF^ are viable and fertile with longer latency for tumor development as compared to p53^-/-^ mice [22]. In contrast to p53 deficiency, loss of p19^ARF^ is supposed to overcome senescence and allow infinite proliferation without gaining malignant properties [25, 26].

In this study we aimed at establishing LSECs from p19^ARF-/-^ mice. LSECs isolated from p19^ARF-/-^ mice, termed mLSECs escape from senescence and are allowed to proliferate without losing overt genetic stability. mLSECs show strong EC characteristics and vascular properties that can be used in homotypic cell-cell interaction studies.

## Materials and Methods

### Ethics

The Ethics Committee for Laboratory Animal Research of the Medical University of Vienna specifically approved this study (Permit Number: BMWF-66.009/0249-II/3b/2012 and BMWFW-66.009/0121-WF-II/3b/2014). All efforts regarding anesthesia and analgesia were made to minimize suffering of animals. In particular, we intraperitoneally injected 100 mg/kg Ketamin and 5 mg/kg Rompun for anesthesia of mice before undergoing liver perfusion. 10 mg/kg Carprofen were subcutaneously injected into mice for analgesia. Prior to euthanasia by cervical dislocation, mice were intraperitoneally injected with 100 mg/kg Ketamin and 5 mg/kg Rompun.

### Isolation of cells

Mouse liver sinusoidal endothelial cells (mLSECs) were isolated from livers of 10–14-weeks-old female p19^ARF-/-^ and C57/BL6 wt mice by *in situ* liver perfusion via the intrahepatic vena cava as described previously [25]. The Ethics Committee for Laboratory Animal Research of the Medical University of Vienna specifically approved this study (Permit Number: BMWF-66.009/0249-II/3b/2012 and BMWFW-66.009/0121-WF-II/3b/2014). All efforts regarding anesthesia and analgesia were made to minimize suffering of animals. The cell suspension was passed through a 70 μm strainer and centrifuged two times at low speed to remove hepatocytes. Supernatants containing the non-parenchymal fraction of liver cells were collected after extensive washing with 0.1% bovine serum albumin (BSA; GE Healthcare, GB, Cat.# K45-001) in phosphate buffered saline (PBS). Cell pellets were resuspended in 17.6% Optiprep solution (PROGEN Biotechnik, Germany, Cat.# 1114542), overlaid by 8.2% Optiprep and 0.1% BSA/PBS prior to centrifugation without brake. The fraction between 17.6% and 8.2% Optiprep containing mLSECs and Kupffer cells was collected, washed and seeded on plastic dishes. While Kupffer cells immediately adhered to culture dishes, LSECs remained floating in the medium. Finally, the medium containing mLSECs was collected and plated on dishes.

### Cell culture

p19^ARF-/-^ and wt mLSECs were cultivated on collagen-coated (Collagen Type I-Rat Tail, BD Biosciences, USA, Cat.#354236) Petri dishes in DMEM (Dulbecco´s Modified Eagle´s Medium) containing 100 μg/ml Endothelial Cell Growth Supplement (ECGS, Biomedical Technologies, USA, Cat.#BT203), 0.2 μg/ml hydrocortisone (Alfa/Aesar, Germany, Cat.#A16292) and 50 μg/ml heparin (AppliChem, Germany, #3U009511). Human hepatic sinusoidal endothelial cells (hHSECs) (ScienCell, USA, Cat#5000) were cultivated in Endothelial Cell Medium (ECM) (ScienCell, USA, Cat. #1001) following the manufacturer’s instruction. Human telomerase reverse transcriptase (TERT)-immortalized blood endothelial cells (BECs; [27] were cultivated on collagen-coated plates in ECM. MIM1-4 cells were cultivated as described previously [25]. All cell lines were cultured at 37°C and 5% CO_2_ and routinely screened for the absence of mycoplasma.

### Proliferation kinetics

5 x 10^3^ mLSECs were seeded in triplicates on collagen-coated 24-well plates and allowed to proliferate for 12 days until cells undergo growth arrest due to high cell density. The number of cells in the corresponding cell populations was determined in a multichannel cell analyzer (Casy Cell Counter, Schärfe Systems, Germany). In addition, a cumulative growth analysis which allows monitoring of proliferation kinetics without an arrest by cell density was performed as described previously [28]. Briefly, 1 x 10^5^ cells were seeded on collagen-coated 6 well-plates. The number of cells was determined periodically in a multichannel cell analyzer (Casy Cell Counter, Schärfe Systems, Germany) until day 12. Cumulative cell numbers were calculated from the cell counts plus dilution factors.

### Immunofluorescence

2 x 10^4^ cells were seeded on collagen-coated coverslips to reach confluence. Cells were washed with PBS and fixed in 4% paraformaldehyde. After blocking with 5% BSA/PBS, incubation with primary antibodies against ZO-1 (rabbit polyclonal-anti-ZO-1, Zymed, USA, Cat#40–2300, Lot#481728A), β-catenin (mouse monoclonal anti-ß-catenin, BD Transduction Laboratories, USA, Cat.#610154, Clone: 14), N-cadherin (mouse monoclonal anti-N-cadherin, USA, Cat.#610920, Clone: 32) and vimentin (mouse monoclonal anti-vimentin, Sigma, USA, Cat.#v5255, Lot# vim 13.2) were employed at a dilution of 1:100. Samples were washed and further incubated with corresponding secondary antibodies. Nuclei were counterstained with 4',6-diamidino-2-phenylindole (DAPI).

### Immunoblotting

Western blot analysis was performed as described previously [29]. Primary antibodies against CD31 (rabbit polyclonal, Abcam, GB, Ab28364), VE-cadherin (rabbit polyclonal, Abcam, GB, Ab33168) and VEGFR-2 (rabbit monoclonal, Cell Signalling, USA, #24795) were used at a dilution of 1:500, while anti-actin (rabbit polyclonal, Sigma-Aldrich, USA, A2066) was employed at 1:3000. Horseradish peroxidase (HRP),biotinylated secondary antibodies and HRP-coupled streptavidin (Invitrogen, #434323, California, USA) were used for detection. Human hepatic sinusoidal endothelial cells (HSECs) and human TERT-immortalized blood endothelial cells (BECs) served as positive controls, whereas hepatocytes isolated from p19^ARF-/-^ mice (MIM1-4) were used as a negative control. Actin was used as a loading control.

### Quantitative reverse-transcriptase polymerase chain reaction (qRT-PCR)

Total RNA was extracted, treated with DNaseI and reverse transcribed using a RNA isolation and cDNA synthesis kit as recommended by the manufacturer (Quiagen, Hilden, Germany). Aliquots of cDNA were employed for Fast SYBR green qPCR (Applied Biosystems, Foster City, USA) 2 and quantified with the 7500 Fast Real-Time PCR System (Applied Biosystems, Foster City, USA). The following probes, forward and reverse primer sequences were used: CD32b: 5’- CCATCTGGACTGGAGCCAAC-3’ and 5’- TGGCTTGCTTTTCCCAATGC-3’; CD146: 5’-CGGGTGTGCCAGGAGAG-3’ and 5’-ACCAGTCCACTTGGCTGAAG-3’; vWF: 5’-TGGCAAGGTTTTTCAGGGGA-3’ and 5’-TAAGCAGGTGATGCAGAGGC-3’; Stab-1: 5’-ACGCTTCTAACGCCACCTTT-3’ and 5’-CCACACGATGACGTGGCTAA-3’; Stab-2: 5’-CACTATGTCGGGGATGGACG-3’ and 5’-GGGAGCGTAGGTGGAATACG-3’; LYVE-1: 5’-TACAGGACCCATGGCTGAGA-3’ and 5’-GGTGCCAAGCATTTCGGTTT-3’; RhoA: 5’-CCATCATCCTGGTTGGGAAT-3’ and 5’-CCATGTACCCAAAAGCGC-3’.

### Tube formation assay

1.2 x 10^5^ cells were seeded in 200 μl Matrigel (9,7μg/ml) (BD Biosciences, USA, #356237) on 48-well plates and stimulated with either 40 ng/ml VEGF (recombinant murine VEGF, Peprotech, USA, #450–32) or 100 μg/ml ECGS and kept at 37°C in a humidified incubator. Tube-like structures were observed 2 hours after seeding, reaching the maximum tube density after 3.5 hours.

### Wound healing

1 x 10^5^ cells were seeded on collagen-coated 6-well plates and grown until confluency. A blue pipette tip was used to generate scratches. Images were taken after cultivation for 20 hours in the corresponding medium and analyzed with ImageJ.

### Flow cytometry

Propidium iodide (PI) (Calbiochem, USA, Cat.#537059) and DAPI (4',6-diamidino-2-phenylindole) staining was performed by following the manufacturer’s instructions. The DNA content was analyzed by flow cytometry (FACS Calibur, Becton Dickinson, USA).

### Cell viability assay

3 x 10^3^ cells were seeded on collagen-coated 96-well plates and treated with drugs at different concentrations after 24 hours. Concentrations of 100, 50, 25, 12.5, 6.25, 3.125, 1.5625, 0.78, 0.39, 0.195, and 0.0975 μM were included. After 72 hours, 3-(4,5-dimethylthiazol-2-yl)-2,5-diphenyltetrazolium bromide (MTT) was added and allowed to be metabolized by cells for 5 hours. Subsequently, cells were lysed with dimethylsulfoxide and evaluated by spectrophotometric detection at 570 nm.

### Statistics

Data were expressed as means ± standard deviation. The statistical significance of differences was evaluated using a paired, non-parametric Student’s t-test. Significant differences between experimental groups were *p<0.05, **p<0.01 or ***p<0.005.

## Results

### Loss of p19^ARF^ allows proliferation of mLSECs

**Fig 1:**
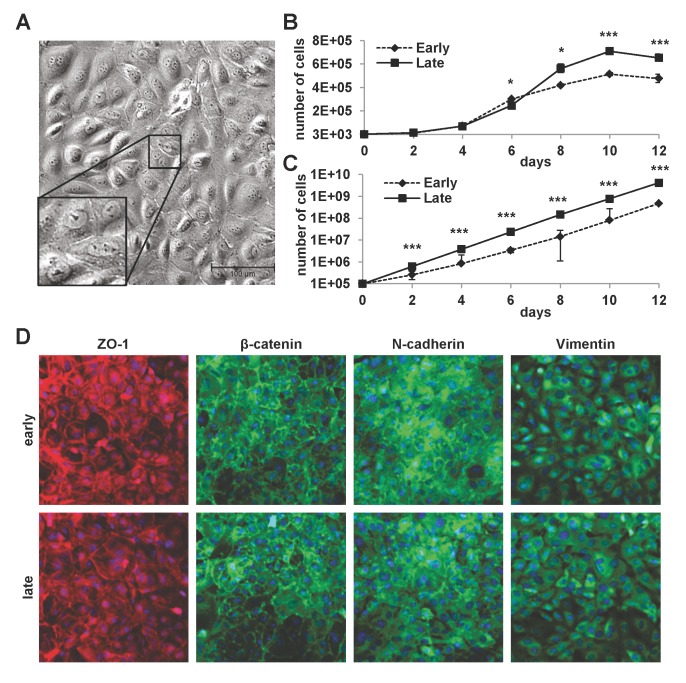
Properties of cultured mLSECs. (A) Phase contrast microscopy of p19^ARF-/-^ mLSECs at passage 5. Inset show cells at higher magnification. (B) Proliferation kinetics by cell density analysis (upper panel) and proliferation kinetics by determination of cumulative cell numbers (lower panel) of mLSECs at early (p5) and late passages (p20). (C) Immunofluorescence analysis showing endothelial junction and intermediate filament proteins expressed in early and late passaged mLSECs. *p<0.05 or ***p<0.005.

mLSECs were isolated from livers of female p19^ARF-/-^ mice. In the presence of the endothelial cell growth supplement (ECGS) medium, cells started to spontaneously proliferate within one week in culture. Phase contrast microscopy showed mLSECs displaying the typical cobblestone-like morphology (Fig 1A). Interestingly, mLSECs showed slightly faster proliferation kinetics at later passages (passage 20) as compared to mLSECs at early passage 5 (Fig 1B). At day 12 of cultivation, mLSECs exhibited proliferation arrest due to high cell density. Similar results were obtained from cumulative growth analyses of mLSECs at early and late passage without proliferation arrest (Fig 1B). Immunolocalization of the tight junction constituent ZO-1 and the adherens junction component β-catenin showed staining at cell boundaries in both early and late passaged mLSECs, indicating cells of epithelial origin (Fig 1D). Similarly, N-cadherin which is required for the interaction among ECs and pericytes was detected at cell borders [30]. In addition, both early and late passaged mLSECs displayed the expression of the intermediate filament protein vimentin which plays an important role in the sprouting of ECs during angiogenesis [31]. Together, these data show that loss of p19^ARF^ equips mLSECs with the ability to proliferate by concomitantly displaying an endothelial morphology.

### mLSECs display endothelial properties in culture

**Fig 2:**
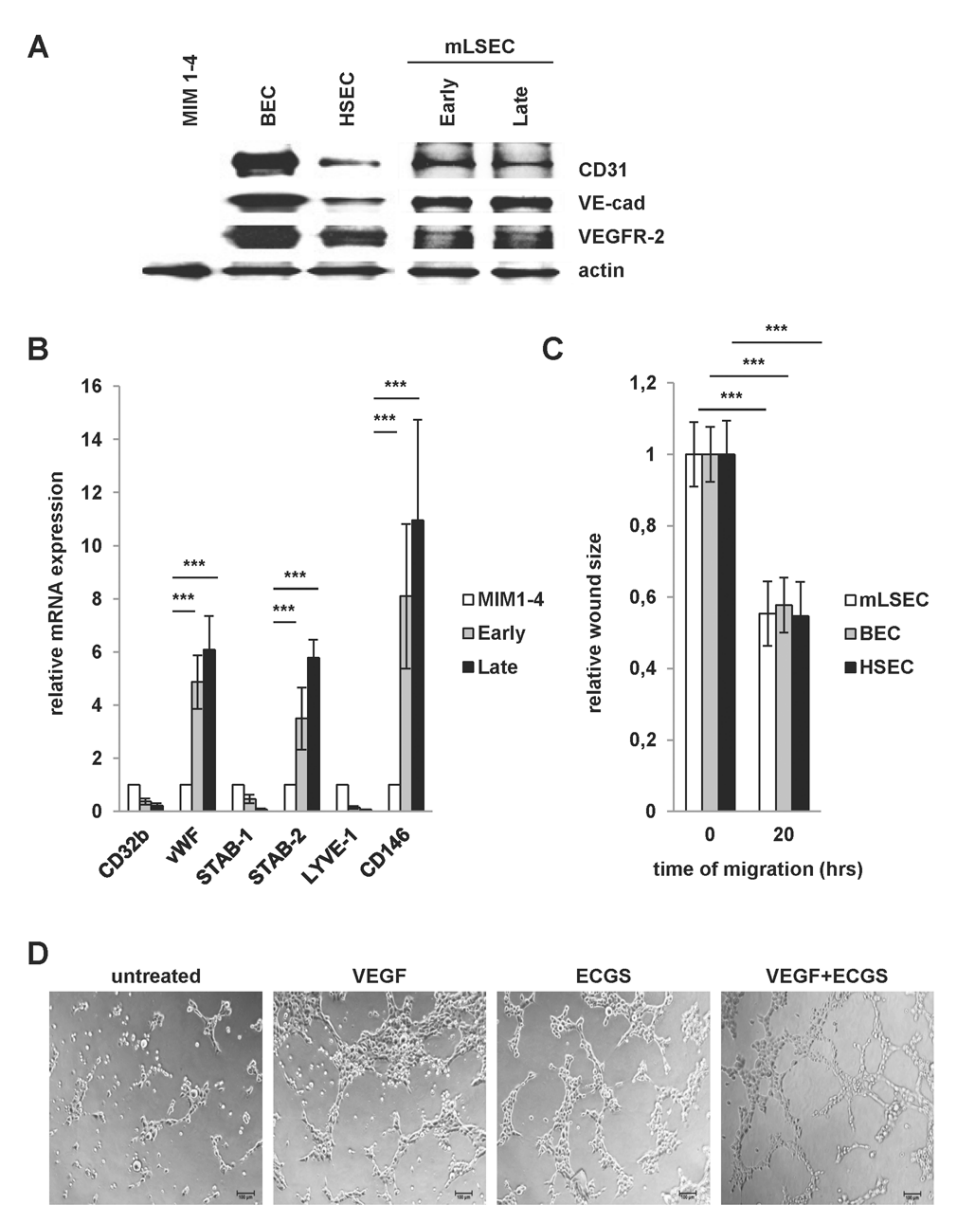
Endothelial integrity of mLSECs. (A) Western blot analysis of early and late passaged mLSECs using anti-CD31, anti-VE-cadherin and anti-VEGFR-2 antibodies. The EC phenotype of mLSECs were compared to blood endothelial cells (BECs) and hHSECs. Immortalized p19^ARF^-deficient hepatocytes, termed MIM1-4, were used as a negative control. Actin is shown as a loading control. (B) qPCR analyses of Stab-1, Stab-2, vWF, Lyve-1, CD32b and CD146 in mLSECs. MIM1-4 cells served as a negative control. RhoA was used as a housekeeping gene. (C) Migratory abilities of mLSECs, BECs and HSECs as shown by wound healing assay in medium containing ECGS. Quantification of phase contrast images of cells after wounding (0 hours) and after wound closure (20 hours). (D) Tube formation of mLSECs as detected after cytokine stimulation.

Next we analyzed more closely the EC phenotype of mLSECs. Immunoblotting showed that mLSECs express CD31, VE-cadherin and VEGFR-2 similarly to hHSECs and the human TERT-immortalized blood endothelial cells (BECs, Fig 2A). Interestingly, mLSECs retained expression of these markers during cell passaging. Furthermore, we analyzed other published markers for their expression in mLSECs. vWF, Stab-2 and CD146 which are reported as characteristic markers of rodent liver endothelium were detected in both, early and late passaged cells. In contrast, Stab-1, Lyve-1 and CD32b expression were not increased in comparison to immortalized MIM1-4 hepatocytes (Fig 2B). In addition, endothelial cells are endowed with a high migratory capacity which precedes angiogenesis *in vivo* and which could be observed by wound healing assays upon stimulation with ECGS. Notably, mLSECs displayed a capability of wound closure which is comparable to established cultures of BECs and hHSECs (Fig 2C). To further demonstrate the endothelial integrity of isolated mLSECs, we performed tube formation assays on growth factor-reduced Matrigel. Formation of vessel-like structures upon stimulation with pro-angiogenic mitogens is a particular feature of all ECs [32–34]. Isolated mLSECs generated tube-like structures upon stimulation with VEGF-A, ECGS or a combination of both, whereas no tubes were detected without stimulation (Fig 2D). From these data we concluded that mLSECs exhibit characteristics that are typical for ECs.

### mLSECs overcome cellular senescence

**Fig 3:**
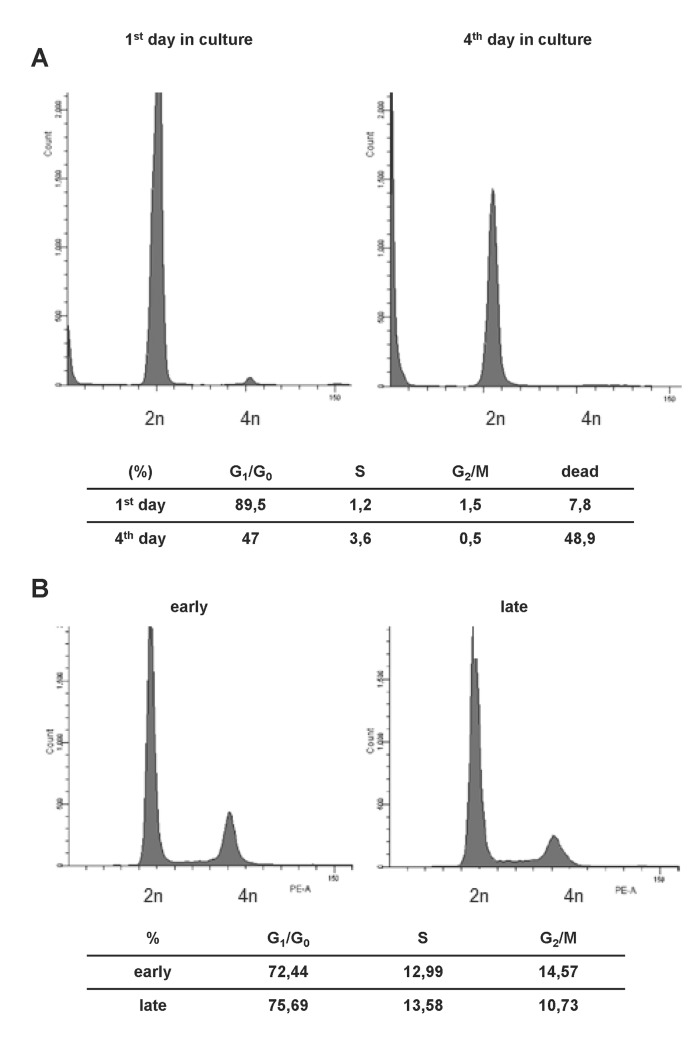
mLSECs represent a genetic stable cell population. (A) Cell cycle analysis of wt mLSECs at day 1 and day 4 post isolation. (B) Cell cycle analysis of early (p10) and late passaged (p20) mLSECs by flow cytometry.

As mentioned, cultivation of rodent LSECs is accompanied by a rapid loss of the endothelial phenotype and onset of cellular senescence several days after isolation [10, 35, 36]. Accordingly, we isolated wild type (wt) LSECs from C57/BL6 mice and introduced them into cell culture. After several days in culture, p19^ARF-/-^ mLSECs started to proliferate (Fig 1A), whereas wt mLSECs did not exhibit any detectable outgrowth under the same culture conditions. Remarkably, cell cycle analyses revealed that 89.5% of freshly isolated wt mLSECs remained quiescent in the G1/G0 phase of the cell cycle at day 1 of cultivation with a low proportion of cell death (7.8%; Fig 3A) indicating an arrest in proliferation. However, the portion of dead mLSECs increased to 48.9% on day 4 after isolation, suggesting that mLSECs evince a clear blockade in cell division followed by cell death (Fig 3A).

The analysis of DNA content by flow cytometry revealed that cultured mLSECs represent a diploid cell population. Higher ploidy levels would point to a non-stable DNA content associated with a malignant phenotype or a contamination with hepatocytes. Interestingly, the cell cycle distribution among different phases of the cell cycle was stable between early (p10) and late (p20) passage numbers, pointing to genomic stability (Fig 3B).

### Loss of p19^ARF^ does not influence susceptibility to anti-angiogenic agents

**Fig 4:**
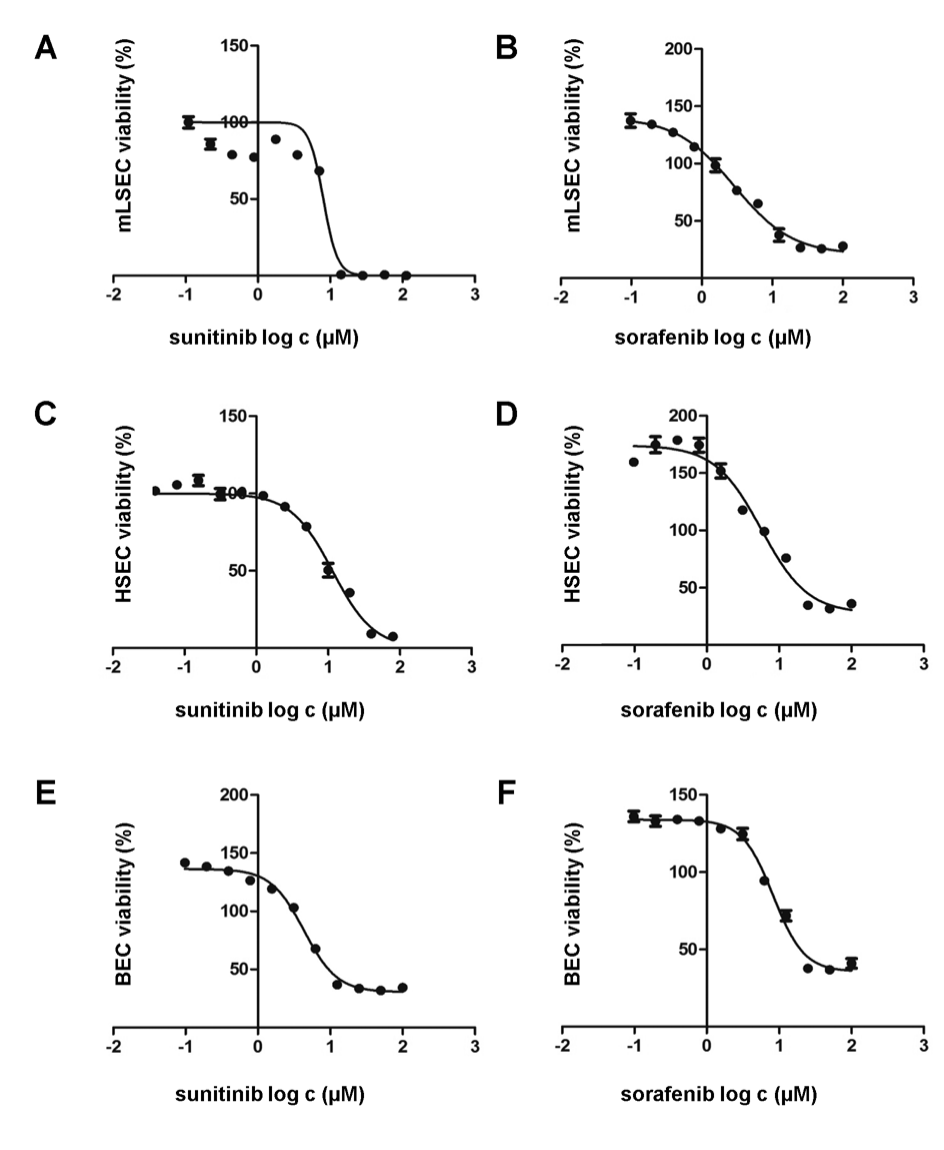
mLSECs respond to angiogenic inhibitors. (A-F) Drug response of mLSECs, hHSECs and BECs to sunitinib and sorafenib as analyzed by MTT assay.

**Fig 5:**
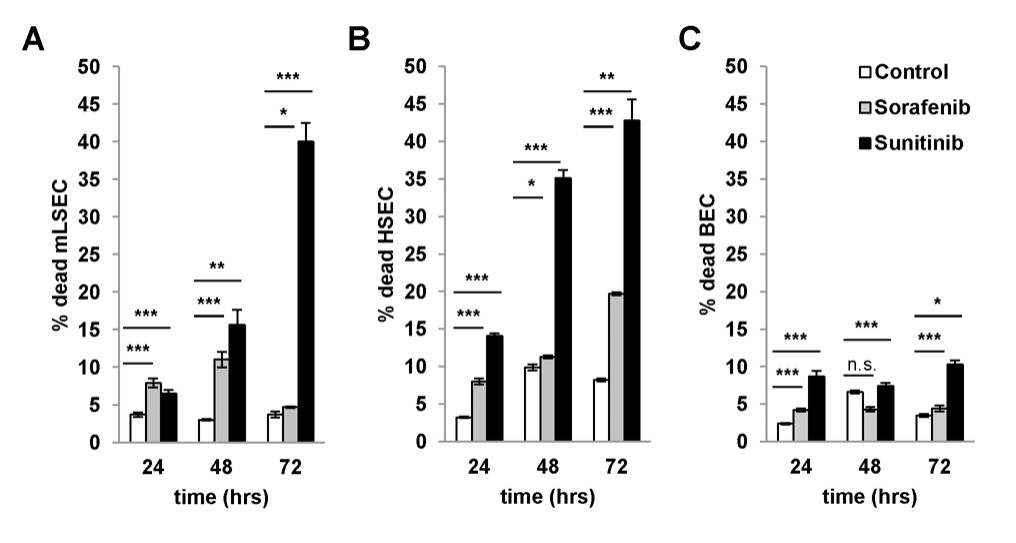
Cytotoxicity and cytostatic effects of angiogenic inhibitors. **(A-C)** Cell cycle analysis by flow cytometry with DAPI staining after treatment of mLSECs, HSECs and BECs with corresponding IC50 values for the times indicated. mLSEC: IC50 sorafenib 7.3 μM, sunitinib 8.0 μM; hHSEC: IC50 sorafenib 2.88 μM, sunitinib 5.67 μM; BEC: IC50 sorafenib 4.33 μM, sunitinib 8.28 μM. *p<0.05, **p<0.01 or ***p<0.005.

We next analyzed whether mLSECs respond to anti-angiogenic drugs such as sorafenib and sunitinib which block platelet-derived growth factor receptor (PDGFR) and VEGFR. Cell survival assays showed similar inhibitory concentration (IC)50 levels of both drugs, namely 8.0 μM for sunitinib and 7.3 μM for sorafenib which are comparable to those detected in BECs (4.33 μM sorafenib; 8.28 μM sunitinib) and hHSECs (2.88 μM sorafenib; 5.67 μM sunitinib), respectively (Fig 4A–4F). To analyze whether the effect of drugs is rather cytostatic or cytotoxic, we performed flow cytometry analysis using DAPI staining. Treatment of mLSECs with sunitinib at the IC50 concentration resulted in 40% of dead cells after 72 hours, thus closely correlating with results from the cell survival assay, while administration with sorafenib led to a cytostatic effect without induction of cell death (Fig 5A–5C). Similar results were obtained by flow cytometry analysis of hHSECs and BECs. These data show that mLSECs respond to anti-angiogenic drugs by inhibition of cellular proliferation and/or cell death.

## Discussion

We have established mouse liver sinusoidal endothelial cells from p19^ARF-/-^ mice. Loss of p19^ARF^ provides the ability to overcome cellular senescence. Isolated mLSECs have a typical cobblestone-like morphology, and in comparison with LSECs isolated from wt mice, mLSECs start to proliferate *in vitro* a few days after introduction into cell culture. By comparing cells at different passage numbers, a slightly faster growth of cells was observed at later passages. As the analysis of proliferation kinetics require seeding of low numbers of cells, we speculate that cells kept longer in cell culture can more easily overcome stress associated with sub-confluent growth and are better adapted to *in vitro* conditions. Notably, mLSECs displayed a stable morphology and kept sensitivity to contact inhibition during cultivation up to passage number 50 (data not shown) which corresponds to roughly 75 cell doublings within 5 months.

mLSECs exhibit an endothelial phenotype due to the expression of typical markers mostly cited in the literature, even if some ambiguity exists among their expression levels. Our study shows that mLSEC express low levels of CD31 which is underlined by the fact that streptavidin/biotin amplification was required to detect the protein. Similarly, VE-cadherin was detected upon signal amplification and its level in hepatic endothelial cells is lower than in BECs. These data support recent reports suggesting that the sinusoidal endothelium in normal liver lacks VE-cadherin or expresses it at low levels and its relative lack might be a consequence of the absence of classical adherens junctions [8]. Further analyses revealed mLSECs expression of vWF as well as Stab-2 and CD146 but not Stab-1, CD32b and Lyve-1, the latter being rather a marker of lymphatic endothelium [37]. Furthermore, mLSECs harbor a high migratory capacity as shown by the wound healing assay as well as tube forming abilities on growth factor-reduced matrigel, reflecting important aspects of endothelial integrity and angiogenic capabilities. In addition, loss of p19^ARF^ does not induce malignancy as shown by subcutaneous injection of mLSECs into immunocompromized mice. None of the animals developed tumors or died 6 months after injection (data not shown), demonstrating that mLSECs fail to exhibit a malignant phenotype.

Cellular senescence is a state of irreversible growth arrest that can be induced in stress response to various cellular stimuli [21]. p19^ARF^ is a known executor of senescence in cultured cells [38] and its expression level is increasing during aging [39]. Thus, loss of p19^ARF^ allows overcoming cellular senescence which was described for several cell types in culture [25, 26]. The mechanism of cell death of primary rodent wt LSECs is still poorly understood and many authors refer to dedifferentiation and loss of phenotype instead of the induction of cellular senescence. We speculate that the upregulation of p19^ARF^ involves the activation of p53, causing the induction of the cell cycle regulators p21^Cip1^ and p27^Kip1^ and thus cell cycle arrest, which subsequently leads to the loss of the EC phenotype and cell death in culture. In accordance with this consideration, loss of p19^ARF^ allows overcoming cell cycle arrest (Fig 3B), which is typical for many primary cells and precedes cellular senescence. mLSECs lacking p19^ARF^ reveals a distribution across all cell cycle phases in early and late passaged cells. In contrast, wt LSECs remain quiescent in the first days of cultivation with about 90% of cells arrested in the G_1_/G_0_ phase of the cell cycle (Fig 3A). On day 4 of cultivation, this portion is reduced to 50% of cells with the other 50% being apoptotic. Noteworthy, treatment of mLSECs with either sunitinib or sorafenib showed that mLSECs are able to respond to anti-angiogenic drugs in a similar way as HSECs and BECs as shown by comparable IC50 levels. Loss of p19^ARF^ still allows mLSECs to respond by cell cycle arrest and cell death that is probably driven by p53 in a p19^ARF^-independent way.

Together, our results show that loss of p19^ARF^ supports the escape of mLSECs from cellular senescence and enables their cultivation *in vitro*. mLSECs can be immortalized without overt features of dedifferentiation. As compared to previously reported p19^ARF-/-^ hepatocytes [25] and p19^ARF-/-^ stellate cells [26], mLSECs do not evince signs of malignancy despite an “unlimited lifespan” and thus could represent a valuable tool in vascular biology and liver-specific studies.

## Abbreviations

BEC: blood endothelial cells
BSA: bovine serum albumin
EC: endothelial cells
ECGS: Endothelial Cell Growth Supplement
hHSEC: human hepatic sinusoidal endothelial cell
IC: inhibitory concentration
HUVEC: human umbilical vein endothelial cells
LYVE: lymphatic vessel endothelial hyaluronan receptor
mLSEC: mouse p19^ARF-/-^ liver sinusoidal endothelial cell
PBS: phosphate-buffered saline
Stab: stabilin
TERT: telomerase reverse transcriptase
VE-cadherin: vascular endothelial cadherin
VEGF: vascular endothelial growth factor
vWF: von Willebrand factor
